# Hysteroscopic Endometrial Defect Following Adenomyomectomy and Incidence of Placenta Accreta Spectrum and Uterine Rupture Complications for Subsequent Pregnancy

**DOI:** 10.1007/s43032-024-01758-7

**Published:** 2025-01-06

**Authors:** Mari Ichinose, Takayuki Iriyama, Osamu Hiraike, Seisuke Sayama, Ayako Hashimoto, Kensuke Suzuki, Mitsunori Matsuo, Masatake Toshimitsu, Takahiro Seyama, Kenbun Sone, Keiichi Kumasawa, Yasushi Hirota, Yutaka Osuga

**Affiliations:** Department of Obstetrics and Gynecology, Faculty of Medicine, The University of Tokyo, 7-3-1 Hongo, Bunkyo-Ku, Tokyo, 113-8655 Japan

**Keywords:** Adenomyomectomy, Hysteroscopy, Placenta accreta spectrum, Prenatal counseling, Uterine rupture

## Abstract

Adenomyomectomy, a therapeutic option for women with adenomyosis who wish to preserve their fertility, has been reported to pose a risk of developing placenta accreta spectrum (PAS) and uterine rupture in future pregnancies. However, the specific clinical factors contributing to these occurrences remain elusive. This study aimed to explore the association between hysteroscopic findings after adenomyomectomy and the incidence of PAS in subsequent pregnancies. We conducted a retrospective analysis of 10 patients (11 pregnancies) who had undergone hysteroscopy following adenomyomectomy and had later delivered at our hospital. In 6/10 patients, postoperative hysteroscopy revealed endometrial defects. However, subsequent evaluations confirmed endometrial restoration within 7–21 months, with five patients achieving pregnancy afterward. The only other patient conceived naturally without waiting for endometrial restoration, resulting in uterine rupture from the site of the placenta percreta. The incidence of clinically diagnosed PAS during cesarean section was 100% (1/1) in pregnancies with preconceptional endometrial defects, 20% (1/5) in those with endometrial restoration, and 0% (0/5) in pregnancies without endometrial defects. Similarly, the incidence of pathologically diagnosed PAS was 100% (1/1), 60% (3/5), and 20% (1/5) in these groups, respectively. Thus, endometrial defects were frequently detected after adenomyomectomy and recovered over time, whereas one patient without endometrial restoration developed uterine rupture complicated by PAS. This study demonstrates that while the presence of an endometrial defect identified by postoperative hysteroscopy may be a risk factor for the occurrence of PAS in subsequent pregnancies, allowing sufficient recovery time for the endometrium may help reduce the risk of uterine rupture.

## Introduction

Uterine adenomyosis, a gynecological disorder among women of reproductive age, involves endometrial glandular tissue and interstitium embedding within the muscle layer. Patients present with severe symptoms, including dysmenorrhea, infertility, and recurrent pregnancy loss. Hormonal therapy is effective for adenomyosis; however, it is unsuitable for patients who wish to conceive. The resection of adenomyosis has not been proven to improve fertility compared to that for uterine fibroids, which have a well-defined border with normal muscle; however, it is considered in patients aiming to preserve their uterus and whose dysmenorrhea cannot be treated except by surgical resection.

With the limited reports on the prognosis of pregnancies after adenomyomectomy, uterine rupture, the most serious complication, occurs at a frequency of 3.2–12.5% [1], at the site of the placenta percreta in most pregnancies [2–4]. Adenomyosis is linked with various obstetric complications, such as second-trimester miscarriage, preeclampsia, and placental malposition [5, 6]. Whether adenomyectomy is useful in improving perinatal outcomes and the appropriate type of preconceptional management remain to be explored. Previously, we found lower rates of preterm membrane rupture, preeclampsia, and small-for-gestational-age infants in pregnancies after adenomyomectomy compared to those with adenomyosis; however, the cesarean blood loss was increased, and pathologically diagnosed placenta accreta spectrum (PAS) occurred in 50.0% of pregnancies after adenomyomectomy [7]. PAS encompasses possible complications occurring during pregnancy after adenomyomectomy, ranging from placenta accreta to placenta percreta, ultimately resulting in uterine rupture. This study refers to these conditions collectively as “PAS and uterine rupture complications.” Proper risk assessment is essential for effective preconception counseling and perinatal management. The endometrium plays a critical role in placentation, and surgeries involving incisions into the endometrium—such as operative hysteroscopy, adhesiolysis for Asherman's syndrome, and cesarean section—are associated with a higher risk of developing PAS [8, 9]. Furthermore, adenomyomectomy reportedly results in postoperative blood flow failure in the myometrium and requires time for recovery [10]. Hence, adenomyomectomy may cause endometrial damage not only due to the endometrial incision but also to insufficient blood flow surrounding the endometrium. Hysteroscopy is the most useful and widely used method for endometrial evaluation.

Nevertheless, to date, little is known about the association of preconception factors with the occurrence of PAS and uterine rupture in pregnancies after adenomyomectomy. The purpose of this study was to investigate the association between endometrial findings in hysteroscopy before pregnancy and PAS and uterine rupture complications in subsequent pregnancies. We reviewed the clinical courses of 10 patients delivering at 12 weeks of gestation or later after adenomyomectomy.

## Methods

This study was approved by the Institutional Review Board of the University of Tokyo Hospital, Japan (approval no: 3053–7). It was a single-center, retrospective study that included patients who had undergone at least one hysteroscopy and magnetic resonance imaging (MRI) after adenomyomectomy and who had subsequently become pregnant and delivered after 12 weeks of gestation. Patients with multiple pregnancies, fetal congenital anomalies, or abnormal uterine morphology were excluded. The primary indication for adenomyomectomy in all women was dysmenorrhea. Those desiring to conceive first underwent fertility treatments, including assisted reproductive technology (ART). Adenomyomectomy was performed following repeated failures of ART [7]. Adenomyomectomy was performed via laparotomy, either using the triple-flap method or asymmetric dissection [1]. Patients who underwent complete lesion resection were included in this study. Clinical signs of PAS were defined as placental placement firmly adhered to the uterine wall during cesarean section, resulting in substantial macroscopic residuals. For all pregnancies after adenomyomectomy in this report, the placenta or, if hysterectomy was performed, the placenta and the resected uterus were submitted for histopathologic examination. The pathological diagnosis of PAS was done after delivery when the smooth muscle and chorionic villi were in contact without the decidual membrane and was categorized as placenta accreta, increta, or percreta. The first hysteroscopic examination and MRI were conducted 3 months after adenomyomectomy. If hysteroscopy after adenomyomectomy revealed endometrial defects or areas with poor blood flow, hysteroscopic reexamination was performed approximately 3–6 months later and repeated if the endometrial restoration was incomplete. For patients in whom endometritis was suspected based on hysteroscopic findings, endometrial histology was conducted, and endometritis was diagnosed when CD138-positive cells were detected. Patients in whom endometritis was diagnosed were prescribed doxycycline hydrochloride hydrate. Patients in whom endometrial defects were initially detected postadenomyomectomy but improved to a minimum residual scar after reexamination were considered to have endometrial restoration and were allowed to become pregnant. Patients who conceived without hysteroscopic evidence of endometrial restoration were considered to lack endometrial restoration. The association of endometrial defects with the development of PAS and uterine rupture complications was examined.

## Results

**Fig. 1:**
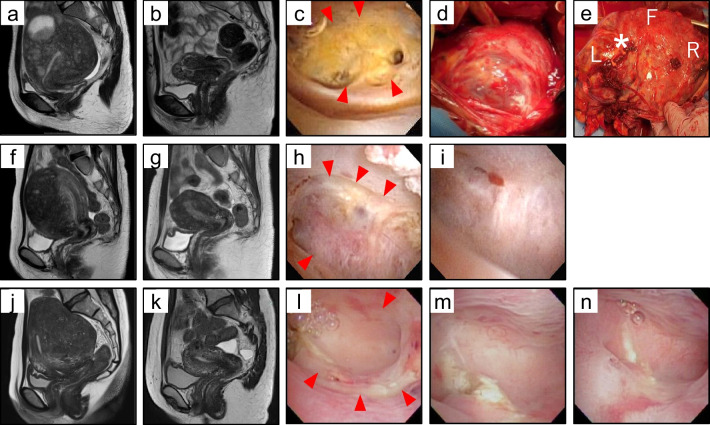
Endometrial defect and restoration after adenomyomectomy. (**a**, **b**) Case 1 magnetic resonance imaging (MRI) before and after adenomyomectomy. (**c**) Hysteroscopic finding in case 1. There was an endometrial defect at the uterine fundus (red arrowheads) 3 months after adenomyomectomy. (**d**) Intraoperative image of case 1 during cesarean section. The placenta is visible through the uterine serosa at the anterior wall of the uterus (white arrowheads). (**e**) Uterine rupture through the percreta observed at the posterior wall of the resected uterus. Multiple hemorrhages were observed through the vessels of percreta in the abdominal cavity during the cesarean section (*). “F,” “R,” and “L” indicate fundus, right, and left, respectively. (**f**, **g**) Case 4 MRI image before adenomyomectomy. (**h**, **i**) Hysteroscopic findings in case 4, endometrial defect at the uterine fundus surrounded by red arrowheads, and restoration 3 and 7 months after adenomyomectomy. (**j**, **k**) Case 5 MRI image before and after adenomyomectomy. (**l**, **m**, **n**) Hysteroscopic findings in case 5, endometrial defect at the uterine fundus surrounded by red arrowheads, and restoration 3, 6, and 8 months after adenomyomectomy

Of the 18 pregnancies delivered after 12 weeks of gestation at our hospital, 11 pregnancies (10 patients) underwent at least one hysteroscopy before pregnancy. The indication for adenomyomectomy was dysmenorrhea for all the patients; among those, patients who wished to conceive first underwent ART (7 patients), and adenomyomectomy was performed after repeated ART failure. There was no case of the second trimester miscarriage. The maternal backgrounds and courses after adenomyomectomy and before pregnancy are summarized in Table 1. All cases involved a partial breach of the uterine endometrium. The median thickness of the thinnest myometrium on MRI taken 3 months postoperatively in all patients was 9.9 (6.7–18.7) mm, and six patients had endometrial defects based on hysteroscopy. The first patient (case 1) had diffuse adenomyosis uteri, with an 88 g adenomyotic lesion resected (Fig. 1a), and an MRI 3 months postoperatively revealed a myometrial layer of 6.7 mm (Fig. 1b). Hysteroscopy revealed an endometrial defect in the uterine fundus, turning yellow with a clear border with the normal endometrium (Fig. 1c). Given these hysteroscopic findings and the presence of CD138-positive cells in the endometrial histology, doxycycline hydrochloride hydrate was prescribed for endometritis, and embryo transfer was postponed so that pregnancy could occur after waiting for endometrial restoration. However, spontaneous pregnancy was achieved without waiting for endometrial restoration. The other five patients (cases 2–6) with endometrial defects after adenomyomectomy were judged to be restored within 7–21 months after adenomyomectomy, permitting pregnancy. In case 4, which was a case of diffuse adenomyosis, a 156-g adenomyosis lesion was resected (Fig. 1f, g). An extensive endometrial defect was detected at the uterine fundus 3 months after adenomyomectomy (Fig. 1h), which recovered to a minimum scar after 7 months (Fig. 1i). Doxycycline hydrochloride hydrate was prescribed for endometritis with implantation failure. In case 5, adenomyomectomy (140 g) was conducted (Fig. 1j, k). Hysteroscopy revealed an extensive endometrial defect at the uterine fundus 3 months after adenomyomectomy. However, the area of the endometrial defect gradually decreased at 6 and 8 months postoperatively, and embryo transfer was performed (Fig. 1l–n).

**Table 1:** List of clinical backgrounds and courses before pregnancy

Case		Findings and clinical courses before pregnancy						
	Age	P	Resected volume (g)	Minimum thickness of uterine wall (MRI) (mm)	Endometrial defect (hysteroscopy)	Endometrial restoration	Doxycycline hydrochloride hydrate for endometritis	Duration (m) adenomyomectomy ~ endometrial restoration
1	35	0	88	6.7	( +)	(-)	( +)	NA
2	42	0	183	18.7	( +)	( +)	( +)	13
3	40	0	100	9.2	( +)	( +)	(-)	11
4	40	0	156	9.6	( +)	( +)	( +)	7
5	42	0	140	12.8	( +)	( +)	(-)	8
6	46	0	30	14.3	( +)	( +)	(-)	21
7	42	0	35	15.3	(-)	NA	(-)	NA
8	44	1	151	12.0	(-)	NA	(-)	NA
9	31	0	25	7.6	(-)	NA	(-)	NA
10	37,40*	0,1*	15	9.9	(-)	NA	(-)	NA

*P* parity, *NA* not applicable, *MRI* magnetic resonance imaging

*Case 10 became pregnant twice after adenomyomectomy

The course of each patient from pregnancy to delivery is depicted in Table 2. All pregnancies after adenomyomectomy were delivered via cesarean section; of the 11 pregnancies, two underwent emergency cesarean sections due to uterine rupture (case 1) and increased uterine contractions (case 4). Pathologically diagnosed PAS was present in five pregnancies, and in two of them (cases 1 and 4), the placenta could not be detached from the myometrium and required hysterectomy. Case 1, in whom conception was achieved with an endometrial defect, showed intraperitoneal bleeding from the site of placenta percreta, resulting in uterine rupture. The uterine wall was extremely thin, resembling a membrane, and the placenta was visible through the anterior wall of the lower uterine serosa (Fig. 1d). Hemorrhages were observed at multiple sites in the placenta percreta on the posterior wall of the uterus (Fig. 1d, e), and cesarean hysterectomy was performed. In case 4, part of the placenta could not be manually detached in a 3-cm^2^ area; hence, a cesarean hysterectomy was conducted, confirmed with a pathological diagnosis of placenta accreta. In the other three cases (cases 2, 3, and 7), the placenta was manually detached with ease; however, placental pathology revealed direct contact of the villi with the smooth muscle, with a diagnosis of placenta accreta. In cases 5 and 6, which became pregnant after the endometrial defect was restored, there was no PAS. PAS did not occur in cases 8–10, wherein endometrial defects were not observed after adenomyomectomy. The median intraoperative blood loss in all pregnancies was 1,520 ml. Two patients who underwent hysterectomy necessitated homologous blood transfusions, while the other six patients underwent autologous blood transfusions. All patients were able to have live babies, and the children were discharged from the neonatal intensive care unit without complications.

**Table 2:** List of clinical courses after pregnancy

Case		Findings and clinical courses after pregnancy							
	Mode of conception	Interval (m) adenomyomectomy to conception	Complications	GW at delivery	Delivery mode	Birth weight (g)	PAS	Hysterectomy	Blood loss (ml)
1	Natural	12	previa	30^+6^	emergencyCS	1629	uterine rupture, percreta	( +)	5380
2	ART	29	previa, GDM	36^+4^	CS	2862	pathological accreta	(-)	1745
3	ART	10	NA	36^+5^	CS	3062	pathological accreta	(-)	920
4	ART	14	SCH, threatened preterm labor	27^+2^	emergencyCS	1109	accreta	( +)	5280
5	ART	15	NA	36^+3^	CS	2450	(-)	(-)	390
6	ART	19	NA	36^+1^	CS	2232	(-)	(-)	1070
7	ART	14	NA	37^+3^	CS	2958	pathological accreta	(-)	2410
8	ART	32	NA	37^+1^	CS	3000	(-)	(-)	2875
9	Natural	10	threatened preterm labor	36^+6^	CS	2780	(-)	(-)	1520
10–1*	ART	21	NA	36^+5^	CS	2258	(-)	(-)	1130
10–2*	ART	62	NA	36^+4^	CS	2718	(-)	(-)	1320

*ART* assisted reproductive technology, *SCH* subchorionic hematoma, *GDM* gestational diabetes mellitus, *CS* cesarean section, *GW* gestational weeks, *NA* not applicable

*Case 10 became pregnant twice after adenomyomectomy

**Fig. 2:**
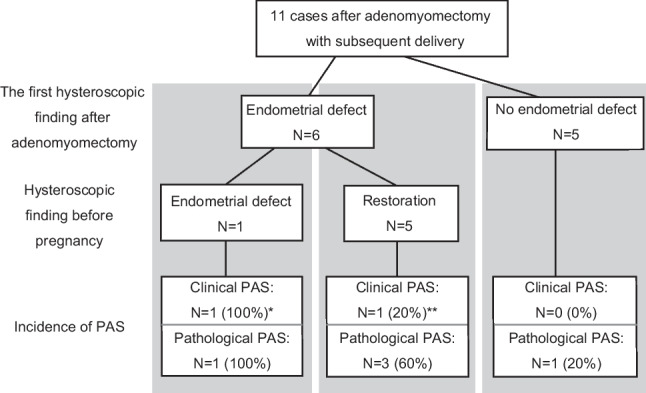
Clinical courses after adenomyomectomy until delivery. The associations between the presence of endometrial defects on postoperative and prepregnancy hysteroscopic findings and the development of PAS in subsequent pregnancies are summarized. Clinical PAS was defined as placental placement firmly adhered to the uterine wall during cesarean section, resulting in substantial macroscopic residuals. Pathological PAS included cases with microscopic findings where the smooth muscle and chorionic villi were in contact without the decidual membrane. All clinical PAS cases were also diagnosed as pathological PAS. *Case 1 in Table 2 that showed uterine rupture through the site of placenta percreta, resulting in hysterectomy. **Case 4 in Table 2 that resulted in hysterectomy due to placenta accreta. CS, cesarean section; PAS, placenta accreta spectrum

Figure 2 illustrates the presence of endometrial defects and the occurrence of pregnancies after restoration. One patient who conceived with an endometrial defect developed a uterine rupture through the placenta percreta site and required a hysterectomy. Among the five pregnancies occurring after repeated hysteroscopies confirmed endometrial restoration, one pregnancy presented with accreta requiring hysterectomy, and the other two pregnancies were pathologically diagnosed as accreta, despite no clinical signs of PAS during cesarean section. None of the five pregnancies without endometrial defects noted on postoperative hysteroscopy showed clinical signs of PAS, whereas one pregnancy was pathologically diagnosed as accreta.

## Discussion

This study is the first to reveal that endometrial defects are frequently detected by hysteroscopy after adenomyomectomy and that postoperative endometrial evaluation may be beneficial in identifying patients at high risk for PAS and uterine rupture complications, enabling optimal management by waiting for endometrial restoration.

Of the pregnancies after adenomyomectomy reported herein, one presented with uterine rupture from the site of placenta percreta, a life-threatening condition for the mother and infant. Two cases of sudden-onset uterine rupture at 27 and 29 weeks of gestation related to placenta percreta have been reported, similar to our experience with case 1 [2, 3]. Uterine rupture is one of the most severe obstetric disorders, often associated with a history of uterine surgery. The frequency is 0.27–0.7% in vaginal deliveries after a previous cesarean section [11], 0.26% in pregnancies after myomectomy [12], and 3.2–12.5% in pregnancies after adenomyomectomy [1], suggesting a particularly high risk of uterine rupture after adenomyomectomy. Since uterine rupture after adenomyomectomy often occurs in PAS, predicting and preventing PAS, especially uterine rupture due to placenta percreta, is of utmost clinical importance.

Hysteroscopic evaluation following adenomyomectomy permitted the assessment of endometrial defects, which may be crucial in predicting uterine rupture in subsequent pregnancies. The extensive endometrial defect led to uterine rupture through the placenta percreta site in case 1. In case 4, where pregnancy was achieved after the extensive endometrial defect recovered to a minimal scar, placenta accreta requiring hysterectomy occurred, indicating that waiting for endometrial restoration could have prevented the severe complications seen in case 1. Of the 11 cases in this study, these two cases (18.2%) were diagnosed as PAS based on clinical intraoperative findings, and both required hysterectomy. The frequencies of clinically diagnosed PAS were 100%, 20%, and 0% for cases with preconceptional endometrial defects, endometrial restoration, and cases without endometrial defects, respectively. Therefore, patients with a history of postoperative endometrial defects are at high risk of PAS, even after endometrial recovery. In cases such as 5 and 6, where pregnancy was avoided while waiting for endometrial restoration, no PAS occurred. Since pathological analysis was performed in all pregnancies, pathological PAS was detected in 100%, 60%, and 20% of pregnancies with preconceptional endometrial defects, cases with endometrial restoration, and cases without endometrial defects, respectively, even if they were not clinically significant. This suggests that a history of endometrial defects might be associated with an increased number of potentially microscopically detected PAS lesions in the background of clinical PAS. Overall, waiting for endometrial healing may reduce the severity of PAS and uterine rupture complications.

PAS involves contact with the myometrium without a decidual membrane. Since the decidual membrane is derived from the maternal endometrium, it is reasonable to assume that placentation will not be successfully accomplished if endometrial defects are recognized prior to conception. There are reports that a history of operative hysteroscopy, adhesiolysis for Asherman’s syndrome, or cesarean section is associated with a high risk of PAS [8, 9]. Additionally, a study on pregnancy after myomectomy, which occurs more frequently after adenomyomectomy, revealed that patients with intraoperative endometrial breaches were significantly more likely to develop PAS than those without [13]. Endometrial injury leads to fibrosis, is reportedly associated with inadequate uterine blood flow, and impedes endometrial healing [14]. In addition to the incision of the endometrium, adenomyomectomy causes temporary insufficiency of blood flow in the surrounding myometrium [10], potentially prolonging healing. This study highlights that insufficient endometrial blood flow may contribute to PAS in subsequent pregnancies. Therefore, it is crucial to develop surgical techniques that prevent postoperative endometrial blood flow insufficiency. We believe that both residual adenomyosis near the endometrium and surgical damage to it can reduce endometrial blood flow in patients who have undergone adenomyomectomy. Our approach focuses on minimizing damage to the endometrial layer while removing as much of the adenomyotic lesion as possible. Preserving endometrial blood flow is likely achieved by minimizing endometrial damage, and maximal removal of adenomyosis makes it easier to reconstruct the uterus by suturing and forming the uterus. We believe that these approaches may support the restoration of uterine blood flow in subsequent pregnancies, but future studies are necessary to determine the relationship between endometrial damage, the extent of resection, and postoperative endometrial blood flow and fertility.

If endometrial defects were observed, hysteroscopy was repeated within 3–6 months. For patients who underwent adenomyomectomy, aggressive hormone replacement for endometrial restoration is challenging due to the risk of adenomyosis recurrence. Hence, allowing pregnancy after endometrial restoration while managing recurrence by taking dienogest may be advisable. In both patients for whom hysterectomy was required due to clinically diagnosed PAS in this report, endometritis was diagnosed before pregnancy, and pregnancy ensued after administering doxycycline hydrochloride hydrate, suggesting the possibility that pregnancy was achieved due to an improved endometrial condition outside the defect. Despite the small number of cases for conclusive findings, qualitative endometrial evaluation, including a history of endometritis treatment alongside hysteroscopic findings, might aid in predicting PAS development.

For predicting pregnancy complications after adenomyomectomy, Otsubo et al. reported that a uterine wall thickness of 7 mm or less on an MRI before pregnancy indicates a high risk for uterine rupture [4]. While they included two patients with uterine rupture related to placenta percreta at 16 and 19 weeks of gestation, uterine rupture after adenomyomectomy was most common at 30–32 weeks of gestation [15]. To date, no report exists on predicting uterine rupture after the second trimester of pregnancy. In our experience, the myometrium was less than 7 mm only in case 1, which presented with uterine rupture, suggesting that myometrial thinning might be beneficial in predicting uterine rupture in the midterm and beyond. Hysteroscopy offers a more detailed view of the endometrium, which is the basis for placentation, and may improve predicting PAS and uterine rupture complications. The weight of the resected adenomyosis, ART, and interval from adenomyomectomy to pregnancy revealed no clear correlation with PAS. Of the 11 pregnancies, nine were IVF-ET pregnancies, and for patients requiring adenomyectomy, embryo cryopreservation before surgery is often planned. In such patients, hysteroscopy can track endometrial changes over time and may be essential in determining the appropriate timing for embryo transfer.

The limitations of this study included the limited number of samples and its retrospective design. Since hysteroscopy was not conducted in patients where adenomyomectomy was performed at other hospitals, these patients were not included despite being perinatally managed at our institution. The duration needed for endometrial restoration and optimal treatment during this period require further study through the accumulation of more cases. Additionally, many patients attempting conception postadenomyomectomy are of advanced maternal age, necessitating individual determination of the waiting period for endometrial restoration through shared decision-making in prenatal counseling. Extending the period of contraception post-surgery can lead to significant psychological stress. Providing preconception counseling about the importance of waiting for endometrial restoration while considering the risk of PAS prior to adenomyomectomy helps patients better understand the treatment plan, alleviating anxiety and promoting mental resilience throughout the process.

Adenomyectomy is an essential treatment option for patients who wish to preserve their fertility. Patients should be thoroughly counseled about perinatal risks preoperatively, and postoperative endometrial evaluation via hysteroscopy may aid in determining the optimal timing for conception to mitigate the risk of PAS and uterine rupture complications.

## Data Availability

Data is available upon request to the corresponding author.
